# PATH classification: a proposal for patients with HNSCC treated with salvage surgery

**DOI:** 10.1007/s00405-024-08961-x

**Published:** 2024-09-23

**Authors:** Albert Llansana, David Virós Porcuna, Rosselin Vasquez, Arnau Parellada, Cristina Valero, Anna Holgado, Xavier León

**Affiliations:** 1 Otorhinolaryngology Department, Hospital Mutua de Terrassa, Universitat Autonoma, Terrassa, Spain; 2 Otorhinolaryngology Department, Hospital Vall Hebron Campus, Universitat Autònoma de Barcelona, Passeig Vall d’Hebron, 119-129, Barcelona, 08035 Spain; 3 Otorhinolaryngology Department, Hospital de la Santa Creu i Sant Pau, Universitat Autònoma de Barcelona, Barcelona, Spain; 4 Centro de Investigación Biomédica en Red de Bioingeniería, Biomateriales y Nanomedicina (CIBER-BBN), Madrid, Spain; 5 UVIC, Universitat Central de Catalunya, Vic, Spain

**Keywords:** Head and neck cancer, Recurrent disease, Salvage surgery, rpTNM, Postoperative prognostic classification

## Abstract

**Purpose:**

The aim of this study is to propose a classification for patients with recurrent head and neck squamous cell carcinoma (HNSCC) treated with salvage surgery based on the location of the primary tumor and data commonly found in the pathological report of the resection.

**Methods:**

Retrospective study of 665 patients with HNSCC treated with a salvage surgery after a local and/or regional recurrence of the tumor.

**Results:**

We propose a new postoperative classification for patients with recurrent HNSCC treated with salvage surgery. PATH classification stratifies patients into 4 stages based on the glottic or non-glottic location of the primary tumor, the local and regional pathologic extension of the tumor, the status of the surgical margins, and the presence of lymph node metastases with extracapsular spread. The PATH classification was more homogeneous in the prognosis of patients included in each of its stages, and it had a better prognostic discrimination capacity between stages than the rpTNM classification. According to the PATH classification, the 5-year disease-specific survival was: PATH I (*n* = 306) 82.8%; PATH II (*n* = 119) 47.1%; PATH III (*n* = 202) 24.4%; PATH IV (*n* = 38) 3.7%. For the rpTNM classification, the 5-year disease-specific survival was: stage I (*n* = 119) 85.1%; stage II (*n* = 134) 68.4%; stage III (*n* = 111) 59.5%; stage IV (*n* = 301) 33.3%.

**Conclusion:**

The PATH classification for HNSCC patients with local and/or regional recurrence treated with salvage surgery had a better prognostic capacity than the rpTNM classification.

**Level of evidence:**

Level IV.

**Supplementary Information:**

The online version contains supplementary material available at 10.1007/s00405-024-08961-x.

## Introduction

Salvage surgery is often the treatment of choice after a local and/or regional recurrence in patients with head and neck squamous cell carcinoma (HNSCC). The oncologic outcomes achieved with salvage surgery are poorer compared to those obtained with equivalent surgery in the setting of primary disease, with an increased frequency of postoperative complications.

Goodwin [1] conducted a meta-analysis of 32 studies involving 1,633 HNSCC patients treated with salvage surgery between 1980 and 1998, finding a 2-year disease-free survival rate of 51% and a 5-year overall survival rate of 39%. Elbers [2] et al. later performed a meta-analysis of advanced-stage HNSCC patients initially treated with radiotherapy or chemo-radiotherapy, mostly from studies published after 2000, and found a 5-year overall survival rate of 37%. Zenga et al. [3]. conducted a systematic review of patients treated with surgery and adjuvant radiotherapy, reporting a 5-year overall survival rate ranging from 10 to 40%, depending on the study.

Assessing the true extent of recurrence is challenging due to tissue changes from prior surgery and/or radiotherapy. Zbären et al. [4]. found that recurrent laryngeal carcinoma is typically more infiltrative and multifocal, spreading beyond the initially treated area, with higher rates of perineural invasion (PNI), lymphovascular invasion (LVI), and extracapsular spread (ECS) [5].

Differentiating between post-treatment changes and recurrent disease is often difficult, leading to the pathological assessment (rpTNM) usually indicating more extensive disease and providing a better prognosis than the pre-operative clinical assessment (rcTNM).

The aim of the present study is to propose a classification for HNSCC patients with a loco-regional recurrence treated with salvage surgery based on the location of the primary tumor and findings commonly reported in pathological reports such as the local and regional extension of the tumor, the status of the surgical resection margins, and the presence of lymph node metastases with ECS, and to compare the prognostic capacity of this classification with the rpTNM staging.

## Materials and methods

The clinical data used in this study were obtained retrospectively from a database that prospectively collects epidemiological, therapeutic and follow-up information of all patients with a HNSCC treated at our center since 1985 [6].

We included patients with HNSCC located in the oral cavity, oropharynx, hypopharynx or larynx, who had local and/or regional recurrence of the tumor, and who were treated with salvage surgery with radical intention during the period 1985–2020. A total of 681 patients were treated with salvage surgery during the study period. We excluded 12 patients who, in the absence of a new recurrence of the tumor after salvage treatment, did not have a minimum follow-up period of 2 years, and 4 patients in whom the pathological report did not specify the presence of ECS in the performed neck dissection. The present study was carried out on 665 patients treated with salvage surgery who had full information in the pathologic report, and with a follow-up period of more than 2 years.

Once the recurrence was diagnosed, all patients were evaluated by an Oncologic Committee that proposed salvage treatment according to the extent of the recurrence and the characteristics of the patients. Table 1 shows the characteristics of the patients included in the study. Given the interaction between tobacco and alcohol consumption, we proceeded to create a combined variable of toxic consumption with the following categories: no consumption; moderate consumption (< 20 cigarettes/day and/or < 80gr alcohol/day); and severe consumption (≥ 20 cigarettes/day or ≥ 80gr alcohol/day). We included the clinical category corresponding to the primary tumor (cTNM) in use at the time of the diagnosis of the tumor. For the assessment of the pathologic extension of the recurrence at regional level (rpN), the patients were reclassified according to the criteria of the 8th edition of the TNM [7].

**Table 1:** Characteristics of the patients included in the study (RT: radiotherapy; CT-RT: chemo-radiotherapy)

		*N*
Sex	Male	605 (91.0%)
	Female	60 (9.0%)
Age	< 50 years	115 (17.2%)
	50–60 years	196 (29.5%)
	60–75 years	269 (40.5%)
	> 75 years	85 (12.8%)
Tobacco	No	56 (8.4%)
	≤ 20 cigarettes/day	114 (17.1%)
	> 20 cigarettes/day	495 (74.5%)
Alcohol	No	144 (21.7%)
	≤ 80 g/day	280 (42.1%)
	> 80 g/day	241 (36.2%)
Toxics consumption	No	47 (7.1%)
	Moderate	107 (16.1%)
	Severe	511 (76.8%)
Year of diagnostic	1985–2000	348 (52.3%)
	2001–2010	199 (29.9%)
	2011–2020	118 (17.7%)
Location	Oral cavity	110 (16.5%)
	Oropharynx	103 (15.5%)
	Hypopharynx	46 (6.9%)
	Supraglottis	138 (20.8%)
	Glottis	268 (40.3%)
Type of tumor	Tumor index	611 (91.9%)
	2nd neoplasm	54 (8.1%)
Initial cT	cT1	228 (34.3%)
	cT2	221 (33.2%)
	cT3	170 (25.6%)
	cT4	46 (6.9%)
Initial cN	cN0	479 (72.0%)
	cN1	65 (9.8%)
	cN2	109 (16.4%)
	cN3	12 (1.8%)
Tumor grade	Well differentiated	88 (13.2%)
	Moderately differentiated	525 (78.9%)
	Poorly differentiated	52 (7.9%)
Initial local treatment	Surgery	111 (16.7%)
	Surgery + RT/CT-RT	38 (5.7%)
	RT / CT-RT	516 (77.6%)
Initial regional treatment	Observation	241 (36.2%)
	Surgery	123 (18.5%)
	Surgery + RT/CT-RT	301 (45.3%)
Initial induction chemotherapy	No	492 (74.0%)
	Yes	173 (26.0%)
Disease-free interval	< 6 months	100 (15.0%)
	6–12 months	207 (31.1%)
	> 12 months	358 (53.9%)
Type of recurrence	rT	427 (64.2%)
	rN	138 (20.8%)
	rT + rN	100 (15.0%)

For all the patients included in the study, information was available on the local (rpT) and regional (rpN) pathologic extent of the tumor, the status of the surgical resection margins, and the presence of ECS in case of regional involvement. A positive surgical margin was considered when there was invasive cancer present at the edge of the specimen, and a close surgical margin when invasive cancer was less than 5 mm from the edge. A total of 138 patients initially cN0 and without clinical evidence of lymph node involvement at the time of local salvage surgery were treated with an elective neck dissection, which was positive on 25 occasions (18.1%). Patients treated only with local tumor resection without lymph node surgery were included in the rpN0 group of patients. Patients treated only with salvage neck dissection were classified as rpT0.

Before salvage surgery, 3.5% of patients with isolated local recurrence (*n* = 15), 15.9% of patients with isolated regional recurrence (*n* = 22), and 9.0% of patients with loco-regional recurrence (*n* = 9) were treated with chemotherapy. The indication for chemotherapy was carried out on an individualized basis in patients with a good general condition and with advanced local and/or regional recurrence in whom it was considered that a reduction in tumor volume would facilitate the salvage surgery.

A total of 153 patients (23.0%) received adjuvant treatment with radiotherapy (*n* = 108) or chemo-radiotherapy (*n* = 45). The percentage of patients who received adjuvant treatment after an isolated local recurrence was 6.8% (*n* = 29), after an isolated regional recurrence it was 67.4% (*n* = 93), and after a loco-regional recurrence it was 31.0% (*n* = 31). Table 1 of the Supplementary Material shows the distribution of adjuvant treatment with radiotherapy or chemo-radiotherapy according to the type of recurrence. The indication for adjuvant treatment was decided on an individual basis by the Oncologic Committee considering the pathologic extent of the recurrence, previous treatment, and the general condition and characteristics of the patient. In general, those patients with positive surgical margins and/or lymph node metastases with ECS were considered candidates for adjuvant treatment. In all cases, an assessment of previous radiotherapy treatment was carried out in order to adjust the dose and irradiation fields.

Survival estimates were performed from the date of salvage surgery. The mean follow-up period for patients after salvage surgery was 5.5 years (standard deviation 5.9 years). Four patients died as a result of complications associated with the salvage surgery. These patients were considered deceased as a consequence of the tumor for the purpose of survival estimation.

Fifty-four patients had additional salvage surgery for a new local (*n* = 41) or regional (*n* = 13) recurrence of the disease. Only the first salvage surgery was considered in the present study.

We performed a recursive partitioning analysis considering as dependent variable the disease-specific survival and as independent variables the primary location of the tumor, and pathologic outcomes including the local (rpT) and regional (rpN) extension of the tumor, the status of the surgical resection margins, and the presence of lymph node metastases with ECS.

In the recursive partitioning analysis we used the classification and regression tree method. From the result of the recursive partition analysis, a prognostic classification was defined based on the pathological variables and the primary location of the tumor, which we called PATH (pathological) classification. Survival estimates were made with the Kaplan-Meier method, using the log-rank test in the comparison of the survival curves.

The prognostic capacity of the PATH classification was compared with the pathologic classification according to the 8th edition of the TNM (rpTNM) [7]. In order to objectively compare both classifications, we used the hazard discrimination and balance parameters proposed by Groome et al. [8]. Hazard discrimination measures how evenly spaced the survival curves are for each of the stages of the classification and how large the difference in survival is between the best and worst stage. Hazard discrimination ranges from 0 to 100%, where 100% represents an ideal classification with complete coverage of the survival area by evenly spaced curves. Balance quantifies the distribution in the number of patients included in each of the stages of the classification system. The balance value ranges from 0 to 100%, where 100% represents an ideal classification in which each of the stages has the same number of patients.

The study was approved by the Institutional Review Committee of our center (IIBSP-CCC-2022-99) and it was conducted following the principles established in the Declaration of Helsinki.

## Results

Five-year disease-specific survival after salvage surgery for the patients included in the study was 53.9% (95% CI: 50.0-57.8%), and 5-year overall survival was 43.1% (95% CI: 39.2–47.0%). Table 2 shows the distribution of the patients and the 5-year disease-specific and overall survival according to the location of the primary tumor and pathological variables such as the local (rpT) and regional (rpN) extension of the recurrence, the status of the surgical margins, and the presence of lymph node metastases with ECS.

**Table 2:** Distribution of the patients according to pathologic outcomes and 5-year disease-specific survival (DSS) and overall survival (OS) for each of the categories

		*N* (%)	5-year DSS (95% CI)	*P*	5-year OS (95% CI)	*P*
Location	Oral cavity	110 (16.5%)	41.3% (31.7–50.9%)	0.0001	32.8% (23.8–41.8%)	0.0001
	Oropharynx	103 (15.5%)	33.8% (23.8–43.8%)		19.5% (11.7–27.3%)	
	Hypopharynx	46 (6.9%)	32.7% (18.4–47.0%)		26.1% (13.4–38.8%)	
	Supraglottis	138 (20.8%)	39.8% (31.2–48.4%)		32.8% (24.8–40.8%)	
	Glottis	268 (40.3%)	77.7% (72.4–83.0%)		64.9% (59.0-70.8%)	
rpT	rpT0	138 (20.8%)	39.8% (31.4–48.2%)	0.0001	32.2% (24.2–40.2%)	0.0001
	rpT1	127 (19.1%)	81.4% (74.0-88.8%)		64.5% (55.9–73.1%)	
	rpT2	160 (24.1%)	61.2% (53.4–69.0%)		52.6% (44.8–60.4%)	
	rpT3	103 (15.5%)	52.2% (41.8–62.6%)		38.7% (29.1–48.3%)	
	rpT4	137 (20.5%)	35.8% (27.4–44.2%)		26.3% (18.9–33.7%)	
rpN	rpN0	427 (64.2%)	66.5% (61.8–71.2%)	0.0001	53.5% (48.6–58.4%)	0.0001
	rpN1	35 (5.3%)	51.1% (33.7–68.5%)		39.8% (23.5–56.1%)	
	rpN2	54 (8.1%)	50.4% (36.1–64.7%)		36.7% (23.4–50.0%)	
	rpN3	149 (22.4%)	20.3% (13.6–27.0%)		16.3% (10.2–22.4%)	
Margin*	Negative	384 (72.8%)	69.1% (64.2–74.0%)	0.0001	53.7% (48.6–58.8%)	0.0001
	Close	52 (9.9%)	49.5% (34.4–64.6%)		45.4% (31.1–59.7%)	
	Positive	91 (17.3%)	15.4% (7.8–23.0%)		13.9% (6.6–21.2%)	
Extracapsular spread **	No	69 (29.0%)	55.9% (43.4–68.4%)	0.0001	40.5% (28.5–52.5%)	0.0001
	Yes	169 (71.0%)	21.9% (15.4–28.4%)		18.0% (12.1–23.9%)	

* Only patients treated with a local resection (*n* = 527) / ** Only rpN + patients (*n* = 238)

**Fig. 1:**
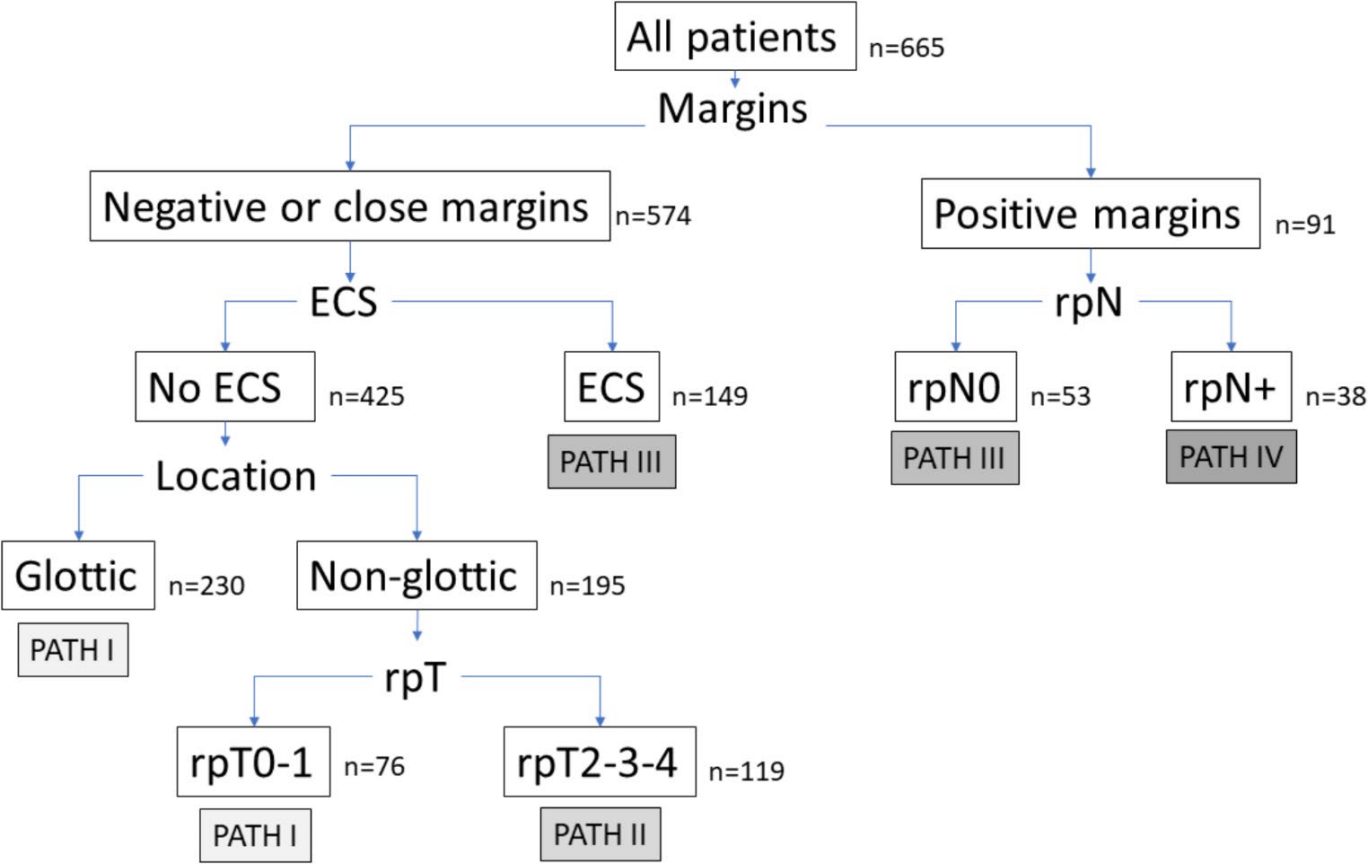
Classification tree obtained with the recursive partitioning analysis (ECS, extracapsular spread)

Figure 1 shows the result of the recursive partitioning analysis considering the disease-specific survival as the dependent variable. We obtained a classification tree with 6 terminal nodes with a first partition at the expense of the surgical resection margins. Patients with positive surgical margins were classified according to the pathological category of regional extension of the tumor (rpN). For patients with negative-close surgical margins or with an exclusive regional recurrence the model included a partition according to the presence of lymph node metastases with ECS. Patients without regional recurrence or with lymph node metastases without ECS were then classified according to the location of the primary tumor. Finally, those patients with a non-glottic tumor were classified according to the pathologic category of local extension of the tumor (rpT). After grouping terminal nodes with similar survival, we defined four prognostic stages, which we called PATH stages (Table 3).

**Table 3:** PATH classification

PATH I	Negative or near margin / No extracapsular spread / Glottic location Negative or near margin / No extracapsular spread / Non-glottic location / rpT0-1
PATH II	Negative or near margin / No extracapsular spread / Non-glottic location / rpT2-3-4
PATH III	Negative or near margin / Extracapsular spread Positive margin / rpN0
PATH IV	Positive margin / rpN+

**Fig. 2:**
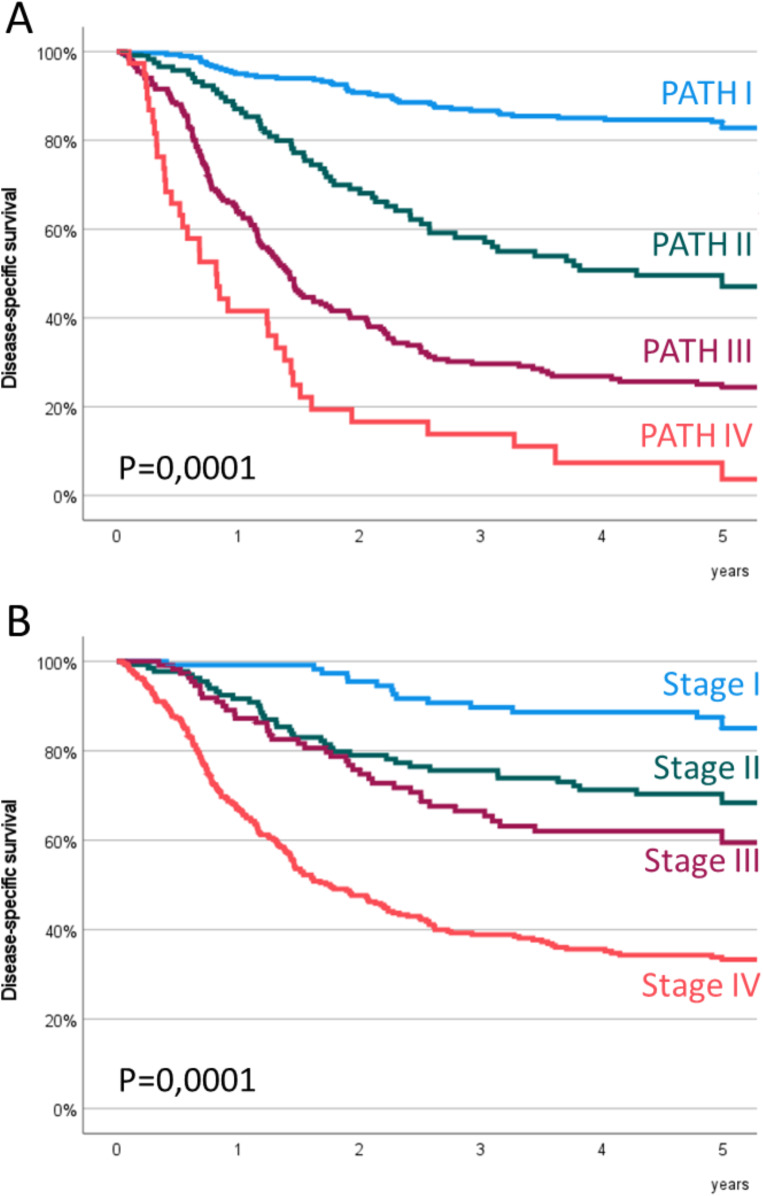
Disease-Specific survival of the patients classified according to the PATH classification (**A**) or the 8th edition of the TNM (**B**)

Figure 2 shows the disease-specific survival curves obtained by applying the PATH (Fig. 2A) and rpTNM (Fig. 2B) classification rules. Table 4 shows the distribution of patients and the corresponding 5-year disease-specific survival according to the PATH and rpTNM classifications.

**Table 4:** Distribution of the patients according to the PATH and rpTNM classification, 5-year disease-specific survival for each category, and Hazard discrimination (Hazard Dis) and balance values corresponding to each classification

		*N* (%)	5-year survival (CI95%)	*P*	Hazard Dis	Balance
PATH	PATH I	306 (46.0%)	82.8% (78.3–87.3%)	0.0001	42.77%	64.76%
	PATH II	119 (17.9%)	47.1% (37.3–56.9%)			
	PATH III	202 (30.4%)	24.4% (18.3–30.5%)			
	PATH IV	38 (5.7%)	3.7% (0.0-10.6%)			
rpTNM	Stage I	119 (17.9%)	85.1% (78.0-92.2%)	0.0001	29.57%	72.81%
	Stage II	134 (20.2%)	68.4% (60.2–76.6%)			
	Stage III	111 (16.7%)	59.5% (49.7–69.3%)			
	Stage IV	301 (45.3%)	33.3% (27.6–39.0%)			

The prognostic quality of the PATH classification was then compared with that obtained by applying the classification rules of the 8th edition of the TNM according to the hazard discrimination and balance criteria proposed by Groome et al. [8]. Table 4 shows the hazard discrimination and balance values for each of the classifications. The Hazard discrimination value was in favor of the PATH classification, while the rpTNM8 classification achieved a more balanced distribution of the number of patients among the different stages.

Table 5 shows the 5-year disease-specific survival for each of the PATH stages according to the rpTNM classification, and for each of the rpTNM stages according to the PATH classification. When applying the PATH classification rules to the rpTNM stages, a highly significant prognostic discrimination capacity was observed for all the rpTNM stages. Notably, patients with rpTNM stage IV, which included the largest number of patients (*n* = 301), were distributed among all PATH categories with an orderly and progressive reduction in specific survival ranging from 74.5% (PATH I) to 4.7% (PATH IV) (*P* = 0.0001).

**Table 5:** Five-year specific survival for PATH stages as a function of rpTNM staging and for rpTNM stages as a function of PATH staging

		*N*	5-year survival (CI95%)	*P*
**rpTNM classification**				
rpTNM stage I	PATH I	111	87.5% (80.8–94.2%)	0.008
	PATH II	0	-	
	PATH III	7	60.0% (17.1–100%)	
	PATH IV	1	0%	
rpTNM stage II	PATH I	71	87.6% (79.6–95.6%)	0.0001
	PATH II	47	49.3% (33.6–65.0%)	
	PATH III	15	40.0% (15.3–64.7%)	
	PATH IV	1	0%	
rpTNM stage III	PATH I	58	77.3% (65.7–88.9%)	0.0001
	PATH II	40	49.1% (32.2–66.0%)	
	PATH III	11	18.2% (0.0-40.9%)	
	PATH IV	2	0%	
rpTNM stage IV	PATH I	66	74.5% (63.1–85.9%)	0.0001
	PATH II	32	40.8% (22.0-59.6%)	
	PATH III	169	21.6% (15.1–28.1%)	
	PATH IV	34	4.7% (0.0-12.9%)	
**PATH classification**				
PATH I	rpTNM stage I	111	87.5% (80.8–94.2%)	0.065
	rpTNM stage II	71	87.6% (79.6–95.6%)	
	rpTNM stage III	58	77.3% (65.7–88.9%)	
	rpTNM stage IV	66	74.5% (63.1–85.9%)	
PATH II	rpTNM stage I	0	-	0.590
	rpTNM stage II	47	49.3% (33.6–65.0%)	
	rpTNM stage III	40	49.1% (32.2–66.0%)	
	rpTNM stage IV	32	40.8% (22.0-59.6%)	
PATH III	rpTNM stage I	7	60.0% (17.1–100%)	0.044
	rpTNM stage II	15	40.0% (15.3–64.7%)	
	rpTNM stage III	11	18.2% (0.0-40.9%)	
	rpTNM stage IV	169	21.6% (15.1–28.1%)	
PATH IV	rpTNM stage I	1	0%	0.638
	rpTNM stage II	1	0%	
	rpTNM stage III	2	0%	
	rpTNM stage IV	34	4.7% (0.0-12.9%)	

In contrast, when applying the rpTNM classification rules to PATH stages, significant differences in survival only appeared for patients with stage PATH III (*p* = 0.044). Patients with stage PATH I, which grouped the largest number of patients (*n* = 306), were distributed across all rpTNM stages, but with no significant differences in survival between stages (*P* = 0.065).

Overall, these results indicate that the PATH classification has a greater ability to group patients with a similar prognosis within each of the stages than the rpTNM classification.

Table 2 of the supplementary material shows the 5-year disease-specific survival according to the PATH classification for each of the head and neck location of the primary tumor. There was an orderly and significant decrease in the disease-specific survival for all locations, except for hypopharyngeal tumors, in which survival of patients with a stage PATH IV (*n* = 3) had a superior survival to that of patients with a stage PATH III (*n* = 22).

## Discussion

According to our results, a classification in which standard parameters included in the pathological reports such as the status of the surgical resection margins or the presence of lymph node metastases with ECS, together with the loco-regional extension of the tumor and the location of the primary tumor, had a better prognostic capacity compared to that obtained by using the pathological classification of the 8th edition of the TNM in patients with a local and/or regional recurrent HNSCC treated with salvage surgery.

Several anatomopathological variables have been related to prognosis in patients with HNSCC treated with salvage surgery. Almost all authors find a significant relationship between positive resection margins and a decrease in survival [9–20]. Other pathological variables that have been related to survival are local extent of recurrence [10, 13, 17, 19, 21, 22], regional involvement [10, 11, 13, 15, 17, 21–25], presence of lymph node metastases with ECS [12, 16, 25], and perineural invasion [9, 10, 25] or lymphovascular invasion [13, 15, 25].

Based on the pathologic findings obtained in patients with HNSCC treated with salvage surgery, Haque et al. [26] defined a high-risk group of patients, which were those who had lymph node metastases with ECS and/or positive margins, and an intermediate-risk group, composed of patients with close margins, lymphovascular or perineural invasion and/or tumor involvement in more than two lymph nodes.

According to the result of a recursive partitioning analysis carried out in our patients, the parameter with the greatest prognostic capacity in patients treated with salvage surgery was the status of the resection margins. From here, the classification criterion for patients with positive margins was lymph node involvement; and in patients with negative or close surgical margins, it was the presence of lymph node metastases with ECS, the glottic or non-glottic location of the tumor, and the category of the local extension of the recurrence.

Several authors have found that patients with extralaryngeal tumors treated with salvage surgery have a poorer prognosis than those located in the larynx [12, 21, 22]. Furthermore, supraglottic tumors have a worse prognosis than those located in the glottis [9]. Similarly, we observed that patients with supraglottic tumors had a significant lower survival than patients with glottic tumors (5-year disease-specific survival for supraglottic versus glottic tumors: 39.8%, CI 95%: 31.2–48.4% versus 77.7%, CI 95%: 72.5–82.9%, *P* = 0.0001). These results justify that in the recursive partitioning analysis the supraglottic tumors were grouped with the extralaryngeal tumors.

One of the main limitations of the rpTNM pathologic classification is that the stages group patients with different prognosis. According to our results, patients with a pathologic rpTNM stage IV (45.3% of our sample), included patients with tumors of relatively good prognosis, such as rpT4 glottic tumors without lymph node involvement and negative resection margins (*n* = 38, 5-year disease-specific survival 79.5%), which were considered as PATH I, together with poor prognostic tumors, such as tumors with positive margins and lymph node involvement, considered as PATH IV. Stage IV in the rpTNM classification included patients with a very disparate prognosis, with 5-year disease-specific survivals ranging from 74.5% (rpTNM stage IV/PATH I patients) to 4.7% (rpTNM stage IV/PATH IV patients).

On the contrary, patients included in each of the PATH classification categories had a much more homogeneous prognosis. The only PATH stage in which significant differences in disease-specific survival appeared when classifying patients according to the rpTNM classification was in PATH III. Notably, patients with PATH I, which included the greatest proportion of patients in our series, were evenly distributed between stages I to IV of the rpTNM classification, but without differences in survival according to rpTNM stages reaching statistical significance (*P* = 0.065).

In addition to maintaining maximum homogeneity in the survival of patients included in each of the stages, one of the objectives of a prognostic classification system is to achieve maximum discrimination between the different stages. In order to objectively evaluate this capacity for discrimination between stages, Groome et al. [8] proposed a parameter called hazard discrimination, which evaluates the difference in survival between the extreme stages and the distribution of survival of the intermediate stages. According to our results, the PATH classification had a higher hazard discrimination score than that obtained with the rpTNM classification (42.77% in PATH versus 29.57% in rpTNM).

Finally, we calculated the balance, which is a measure of the homogeneity in the distribution in the number of patients included in each of the categories of the classification system. In this case, the balance favored the rpTNM classification, which had a more homogeneous distribution in the number of patients in each of the stages (64.75% in PATH versus 72.75% in rpTNM).

The PATH classification demonstrates a significant decrease in disease-specific survival with increasing PATH stages across all tumor locations, except for hypopharyngeal tumors where stage IV showed better survival than stage III, likely due to a small sample size. This confirms the robustness of the PATH classification in maintaining prognostic capacity regardless of tumor location. Janot et al. [27]. found that adjuvant chemo-radiotherapy post-salvage surgery significantly improved loco-regional control and disease-free survival in HNSCC patients initially treated with radiotherapy. Additionally, an open-label phase II trial indicated that adjuvant immunotherapy (nivolumab) post-salvage surgery was well tolerated and improved disease-free survival [28]. The PATH classification helps identify patients with poor prognosis who may benefit from intensified adjuvant treatments or closer follow-up and those with good prognosis for whom salvage surgery alone may suffice. The study’s limitations include its retrospective design, single-institution sample, and evolving treatment methods over time. External validation is necessary to generalize the results and integrate PATH classification into HNSCC prognostic assessments.

## Conclusion

We propose the PATH classification for patients with HNSCC with local and/or regional recurrence treated with salvage surgery based on the location of the primary tumor and pathological variables such as the local and regional pathologic extent of recurrence, the status of the surgical margins, and the presence of lymph node metastases with ECS. The PATH classification had a better prognostic capacity than that obtained by applying the pathologic classification rules proposed in the 8th edition of the TNM.

## Electronic supplementary material

Below is the link to the electronic supplementary material.

Supplementary Material 1

## Data Availability

The data is available on request.
