# Supplementary material for: PATH classification: a proposal for patients with HNSCC treated with salvage surgery
Source: Eur Arch Otorhinolaryngol. 2024 Sep 23;282(2):971–9. doi: 10.1007/s00405-024-08961-x (PMC11805818; doi:10.1007/s00405-024-08961-x)
Supplement: Supplementary file 1 — Supplementary Material 1 [file 405_2024_8961_MOESM1_ESM.docx]

**Supplementary material.**

**Table 1.** Adjuvant treatment after salvage surgery according to the type of recurrence

|  | **Adjuvant treatment** | **N (%)** |
| --- | --- | --- |
| **rT** | No | 398 (93.2%) |
|  | Radiotherapy | 25 (5.9%) |
|  | Chemo-radiotherapy | 4 (0.9%) |
| **rN** | No | 45 (32.6%) |
|  | Radiotherapy | 62 (44.9%) |
|  | Chemo-radiotherapy | 31 (22.3%) |
| **rT + rN** | No | 69 (69.0%) |
|  | Radiotherapy | 21 (21.0%) |
|  | Chemo-radiotherapy | 10 (10.0%) |

**Table 2.** Five-year disease-specific survival (DSS) according to the PATH classification by location of the primary tumor.

|  |  | **N** | **5-year DSS (95% CI)** | **P** |
| --- | --- | --- | --- | --- |
| **Oral cavity** | PATH I | 24 | 87.1% (73.6-100%) | 0.0001 |
|  | PATH II | 23 | 54.2% (33.2-75.2%) |  |
|  | PATH III | 54 | 21.8% (9.8-33.8%) |  |
|  | PATH IV | 9 | 0% |  |
| **Oropharynx** | PATH I | 6 | 71.8% (51.6-92.0%) | 0.0001 |
|  | PATH II | 20 | 32.4% (9.9-54.9%) |  |
|  | PATH III | 44 | 24.3% (11.2-37.4%) |  |
|  | PATH IV | 13 | 7.7% (0.0-22.2%) |  |
| **Hypopharynx** | PATH I | 7 | 83.3% (53.5-100%) | 0.003 |
|  | PATH II | 14 | 44.0% (16.0-72.0%) |  |
|  | PATH III | 22 | 9.6% (0.0-22.1%) |  |
|  | PATH IV | 3 | 33.3% (0.0-86.6%) |  |
| **Supraglottis** | PATH I | 19 | 64.3% (41.2-87.4%) | 0.0001 |
|  | PATH II | 62 | 52.6% (39.1-66.1%) |  |
|  | PATH III | 48 | 22.9% (10.9-34.9%) |  |
|  | PATH IV | 9 | 0% |  |
| **Glottis** | PATH I | 230 | 85.1% (80.2-90.0%) | 0.0001 |
|  | PATH II | 34 | 40.5% (23.8-57.2%) |  |
|  | PATH III | 4 | 0% |  |
|  | PATH IV | 0 | - |  |
